# The Differences in the Flavan-3-ol and Procyanidin Contents of the Japanese ‘Fuji’ and ‘Orin’ Apples Using a Rapid Quantitative High-Performance Liquid Chromatography Method: Estimation of the Japanese Intake of Flavan-3-ols and Procyanidins from Apple as Case Study

**DOI:** 10.3390/foods10020274

**Published:** 2021-01-29

**Authors:** Toshihiko Shoji, Mina Obara, Tadashi Takahashi, Saeko Masumoto, Hironaka Hirota, Tomisato Miura

**Affiliations:** 1 Food Research Institute, National Agriculture and Food Research Organization, 2-1-12, Kannondai, Tsukuba, Ibaraki 305-8605, Japan; mina02@affrc.go.jp; 2 Hirosaki Industrial Research Institute, Aomori Prefectural Industrial Technology Research Center, 1-1-8, Ougi-machi, Hirosaki-shi, Aomori 036-8104, Japan; tadashi_takahashi@aomori-itc.or.jp; 3 Faculty of Food and Agricultural Sciences, Fukushima University, 1, Kanayagawa, Fukushima-shi, Fukushima 960-1269, Japan; saemail@agri.fukushima-u.ac.jp; 4 Apple Cultivation Guidance Division, Japan Agricultural Cooperatives of Tsugaru Hirosaki, 509-1, Waseda, Godai, Hirosaki-shi, Aomori 036-1331, Japan; hirota-hironaka@ja-tu-hirosaki.jp; 5 Institute of Radiation Emergency Medicine, Hirosaki University Graduate School of Health Sciences, 66-1, Hon-cho, Hirosaki-shi, Aomori 036-8564, Japan; tomisato@hirosaki-u.ac.jp

**Keywords:** apple, flavan-3-ols, Fuji, Orin, procyanidins, high-performance liquid chromatography

## Abstract

Previously, we reported that apple polyphenols and their major active compounds, the flavan-3-ols and the procyanidins, can result in various health benefits in animals and humans, according to clinical studies. Here, we developed a rapid method for quantifying flavan-3-ols and procyanidins using high-performance liquid chromatography with fluorescence detection, where we investigated the amounts of flavan-3-ols and procyanidins in the Japanese major apple production centre, the Aomori Prefecture, from 2016 to 2018. The non-bagged ‘Fuji (*n* = 609)’, the bagged ‘Fuji (*n* = 1101)’, and the ‘Orin (*n* = 504)’ apples were evaluated in terms of their differences in flavan-3-ols and procyanidins based on apple variety and the controlled atmosphere storage. The bagging treatments of the ‘Fuji’ apples resulted in significantly higher concentrations of procyanidins, while changes in flavan-3-ols concentrations were not clearly observed by treatment. In addition, ‘Orin’ had a significantly higher concentration of procyanidins than that of ‘Fuji’. In contrast, the controlled atmosphere storage hardly caused any changes in the flavan-3-ol and procyanidin contents. Hence, we present the concentrations of flavan-3-ols and procyanidins in major Japanese apples using the rapid high-performance liquid chromatography method with fluorescence detection.

## 1. Introduction

Several epidemiological studies have suggested that polyphenols present in the diet including fruits promote beneficial effects in human disease [1]. Polyphenols are divided into several subgroups according to their chemical structure including flavonoids being the largest subclass [2]. The structural variations among these polyphenols affect their biological functions and bioavailability. Therefore, the estimation of specific polyphenol intakes may be more important than individual polyphenol intakes for evaluating the health benefits of polyphenols.

The apple fruit (*Malus domestica*) is widely produced globally. Apples are consumed raw and in processed products, such as dried-fruits, juice, cider, brandy, jam, and vinegar. Japan is an important apple-producing country in Asia. Apple consumption reduces the risk of coronary heart disease, which corresponds to the presence of high concentrations of polyphenols in apples [3,4,5]. Apple contains five different structural subclasses, including phenolic acids and flavonoids, which comprise of phenolcarboxylic acids (e.g., chlorogenic acid), anthocyanins (e.g., cyanidin glycosides), flavonols (e.g., quercetin glycosides), dihydrochalcones (e.g., phloridzin), flavan-3-ols (e.g., epicatechin) as well as their oligomer and polymers, and procyanidins [6]. Procyanidins, commonly known as condensed tannins, are oligomers and polymers of flavan-3-ols through the interflavanoid linkage of 4→8 or 4→6 (Figure S1). Chlorogenic acid is the most abundant polyphenol in the apples as a single polyphenol. Among these polyphenols, flavan-3-ols and procyanidins comprise approximately 70% and reflect the main class of the apple polyphenols [7,8]. Apple procyanidin concentrations significantly correlated with antioxidative activity [9,10] and various animal and human clinical studies have suggested that flavan-3-ols and procyanidins are involved in the various health benefits, such as prevention of dementia, cardiovascular diseases, type II diabetes, and cancer [11,12,13,14].

Generally, flavan-3-ols and procyanidins are reduced in the maturation process, which is similar to other apple polyphenols, but anthocyanins in the apple peel can be increased by environmental factors, such as light and temperature [15,16]. However, the profile and contents of apple polyphenols in the peel are largely different from that of the flesh [17,18]. Additionally, The concentrations of flavan-3-ols and procyanidins have been reported to depend on the variety [6,7] and are affected by the production area [19], the growing climate, the crop season, the cultivar management, and the storage [20,21,22,23,24]. After harvesting, the apples are collected, sorted by size and quality, such as skin colour and the soluble solids concentration, and then sold at the market. These polyphenols can deeply affect the overall the apple qualities in terms of agronomical, organoleptic, and nutraceutical characteristics, that influencing the consumer acceptance severely. However, the data reporting the amount of flavan-3-ols and procyanidins in the original varieties in the respective country is very limited, where apples are cultivated in an experimental field. Additionally, there are no databases of flavonoids and polyphenols that include dietary flavan-3-ols and procyanidins in Japan. Therefore, it is important to understand the differences in the flavan-3-ol and procyanidin contents in the apples that were collected by the suppliers to accurately estimate Japanese intake of flavan-3-ols and procyanidins from apples.

Flavan-3-ols and procyanidins have been measured by various colorimetric methods [25] and chromatographic methods [26]. In general, C18 reversed-phase high-performance liquid chromatography (HPLC) is typically used to analyse flavan-3-ols and procyanidins. However, the methods only offer a limited separation of flavan-3-ols and procyanidins from other polyphenols, because many polyphenols and procyanidin isomers are present in apples, resulting in many overlapping peaks of polyphenols and procyanidins on the chromatogram. Recently, normal phase HPLC with fluorescence detection based on separation of the individual flavan-3-ols and procyanidins using a bonded diol stationary phase-based HPLC column has been applied to analyse flavan-3-ols and procyanidins according to the polymerization degree, because they both have a characteristic fluorescence emission, while the other classes of polyphenols have different sensitivities at different wavelengths in the ultraviolet spectra [26,27].

Hence, in the present study, we modified a rapid quantitative method using HPLC with fluorescence detection to determine flavan-3-ols and procyanidins in a large number of apples, which generated more informative results in a shorter span of time. Additionally, we investigated the amount of flavan-3-ols and procyanidins contained in the ‘Fuji’ and ‘Orin’ apples that were produced in the Aomori Prefecture during the 2016 to 2018 seasons. Here, we present the evaluation of flavan-3-ols and procyanidins with their potential health benefits and differences according to the seasons, varieties, bagging treatment, and the controlled atmosphere (CA) storage in Japan.

The purposes of our research were: (i) to measure the contents of flavon-3-ol and procyanidin in major apple varieties ‘Fuji’ and ‘Orin’ in Japan; (ii) to investigate the differences; and (iii) to estimate Japanese intakes of flavon-3-ol and procyanidin by utilizing the results.

## 2. Materials and Methods

### 2.1. Chemicals

The (–)-epicatechin and procyanidin B2 as well as the HPLC-grade acetonitrile and methanol were purchased from Wako Pure Chemicals Ind. (Osaka, Japan), where all the other chemicals, including the organic solvents, were of reagent grade.

### 2.2. Isolation of Individual Oligomer Standards from Apple Extract by Preparative High-Performance Liquid Chromatography (HPLC)

Individual procyanidin standards from monomers to heptamers were obtained by the modified method of Shoji et al. [28]. Briefly, apple procyanidin rich fraction was prepared from apple ‘Fuji’ juice and fractionated according to the polymerization degree by the preparative normal phase chromatography using an Inertsil^®^ PREP-SIL (30 mm × 250 mm i.d., 10 µm particle size) column (GL Science Inc., Tokyo, Japan). A 1.5g sample of the apple procyanidin fraction was dissolved in methanol (2 mL) and applied to the column. The flow rate was 18 mL/min. The methods were used a binary gradient with mobile phase containing hexane/methanol/ethyl acetate. (mobile phase A) 8:3:1 and (mobile phase B) 2:3:1 (*v*/*v*/*v*) in the hexane–methanol–ethyl acetate system. For the first 30 min, the initial eluent was 100% A solvent, followed by a linear gradient from 0 to 100% B for 150 min. The eluate was monitored by the absorbance at 280 nm. The fractions were collected for the following retention times (RT): monomer fraction, RT 26–30 min; dimer fraction, RT 67–73 min; trimer fraction, RT 87–93 min; tetramer fraction, RT 115–122 min; Pentamer fraction, RT 135–142 min; Hexamer fraction, RT 151–158 min; Heptamer fraction L, RT 167–172 min. Each oligomer fractions obtained were concentrated by rotary evaporation at 45 °C and lyophilized. To confirm the purity of individual standard, the conventional normal-phase chromatography was performed by the method of Obara et al. [10].

### 2.3. Apples (Sampling and Storage)

The ‘Fuji’ and ‘Orin’ apple samples were cultivated within the jurisdiction of the Japan Agricultural Cooperatives of Tsugaru Hirosaki (Aomori, Japan) from the 2016 to 2018 seasons, where the ‘Fuji’ and ‘Orin’ apples were collected during their commercial maturity from 20 October to 10 November. In bagging the ‘Fuji’ apple, unripe apple fruits are bagged after thinning according to the cultivar manual of the apple. The bagging treatment of the ‘Fuji’ apples consisted of bagging up to 30 days before harvest. After harvesting, the apples were collected at the sorting factory of the Japan Agricultural Cooperatives of Tsugaru Hirosaki and sorted by quality, such as skin colour and soluble solid concentration, using the non-destructive sorting and grading system with the use of MIQ-2200T (Shibuya Seiki Co., Ltd., Shizuoka, Japan). According to the apple size and quality, every 10 to 12 apples were selected and prepared for immediate freeze-drying. Furthermore, to evaluate the effect of the CA storage, the harvested apples were stored at 0 °C, with 90% relative humidity and conditions of 2.0% to 3.0% O_2_ and 1.5% to 2.5% CO_2_, usually adopted for marketing, for approximately four months.

### 2.4. Sample Preparations

Whole apples were weighed individually and cut meridionally into eight pieces of equal size, in which four diagonally positioned parts were selected for the analytical sample preparation. Subsequently, after excluding apple peel and core, the apple flesh was weighed. After cutting approximately a 1 cm dice, 150 to 200 g of apples were immediately frozen in liquid nitrogen and stored at −80 °C until lyophilisation. Similarly, apple sample without the peel was weighted after excluding the core. The frozen apples were lyophilised at −80 °C using a vacuum freeze drier (FDU-2110, EYELA, Tokyo, Japan) for 5 days. The water content was determined by weighing before and after freeze-drying apple samples. Next, the lyophilised samples were ground in a mechanical Labo Milser crusher (LM-Plus IFM-800, Osaka Chemical Co. Ltd., Osaka, Japan) and the resulting fine powder was stored in the sample tube with the Ageless^®^ Z-100 PKC oxygen absorber (Mitsubishi Gas Chemical Company Inc., Tokyo, Japan) at −30 °C until extraction.

### 2.5. Extractions of the Flavan-3-ols and Procyanidins

The extraction of flavan-3-ols and procyanidins from the lyophilised apple powder was performed using the previously described method [10]. Briefly, prior to the extraction, the apple powder was left at room temperature. The powder was weighed at 0.5 ± 0.02 g and was placed into a 15 mL disposable centrifuge tube. Eight milliliters of the elute of acetone, distilled water, and acetic acid (70:29.5:0.5, *v*/*v*/*v*) was added. Next, the sample mixture was vigorously shaken using a mixer (EYELA cute Mixer CM-1000, Tokyo, Japan) under ambient conditions for 15 min. Tubes were then centrifuged for 10 min at 1000 rpm. Then, the supernatant was transferred to a 25 mL of volumetric flask. Similarly, the extraction procedures were repeated twice and the extracts were collected and filled up to a total volume of 25 mL. The mixture of acetone and water that was used for the extraction of flavan-3-ols and procyanidins allowed the stable extracts to be obtained because of the denaturation of the polyphenol oxidase. Moreover, it has been confirmed that oligomeric and polymeric procyanidins are properly extracted by using this solvent mixture rather than an alcohol solvent, such as methanol [29]. Therefore, no extensive stability testing was performed during the method validation. Finally, the extracts were stored at –30 °C until the HPLC analysis.

### 2.6. Analysis of the Flavan-3-ols and Procyanidins by a Rapid HPLC with Fluorescence Detection

#### 2.6.1. Chromatographic Conditions

The HPLC analysis for the analysis of the flavan-3-ols and procyanidins was performed using a Shimadzu HPLC system (Shimadzu Corporation, Kyoto, Japan) that was equipped with a DGU-20ASR degasser, LC-20AT pump, SIL-20AC auto sample, RF-20AXS fluorescence detector and a CTO-20AC column oven. The column used was an Inertsil^®^ WP300 Diol (i.d. 4.6 mm × 250 mm; 5 µm particle size) purchased from GL Science Inc. The column temperature was held at 35 °C. The mixtures of acetonitrile and acetic acid (mobile phase A, acetonitrile: acetic acid = 98:2, *v*/*v*), methanol, distilled water, and acetic acid (mobile phase B, methanol: water: acetic acid = 95:3:2, *v*/*v*/*v*) were used as mobile phases. Separation of the flavan-3-ols and procyanidins according to the polymerization degree was performed with an increasing gradient of mobile phase B by the method of Obara et al. [10]: an isocratic elution of 7% B for 0 to 3 min, followed by a gradient elution of 30% B for 57 min, and then to 100% B over the next 10 min. The conditions were held at 7% B for the 7 min prior to the restart. The total run time was 77 min. Elution for the rapid method was performed using an isocratic elution of 7% B for 0 to 1.5 min, followed by an isocratic elution of 98% B for 8.5 min. The mobile phase was subsequently returned to the initial conditions (7% B) to re-equilibrate for 10 min. The total run time was 20 min. For the HPLC with fluorescence detection analysis, the apple extracts were filtered through a 0.45 µm polytetrafluoroethylene syringe filter DISMIC^®^-13HP (ADVANTEC^®^, Tokyo, Japan) prior to injection. The sample injection volume was 5 µL, the flow rate was set at 1.0 mL/min. The fluorescence detection of flavan-3-ols and procyanidins was performed with the excitation and emission wavelengths of 230 and 321 nm, respectively.

#### 2.6.2. Preparation of Standard Solutions

The parent stock solution was made up using (–)-epicatechin (0.0450 g) and procyanidin B2 (0.1020 g), which was dissolved in a 100 mL volumetric flask with 50% methanol solution. Further stock solutions of (–)-epicatechin and procyanidin B2 were made through by the dilution of the parent stock solution at seven concentration levels ranging from 4.5 to 45.0, and 10.55–105.5 µg/mL, respectively. The range was defined as the interval between the upper and the lower levels of the analyte within the calibration curve. The stock solutions were stored at −80 °C until the HPLC analysis.

#### 2.6.3. Calibration Carves

Each calibration curves of (–)-epicatechin and procyanidin B2 were obtained by plotting the peak area against the concentrations of the stock standard solutions of known concentrations at seven concentration levels. Each calibration curves were generated using quantitation functions from Shimadzu LabStations software v5.81 SP1 (Shimadzu). Equations generated via linear regression were used to establish concentrations.

#### 2.6.4. Method Validation

##### Selectivity

A high degree of selectivity was achieved by using fluorescence detection with excitation and emission wavelengths that are specific for flavan-3-ols and procyanidins [26], therefore the test of selectivity was not performed.

##### Limits of Detection (LOD) and Limit of Quantification (LOQ)

Limits of detection (LOD) were calculated by obtaining the average area (signal to noise ratio >3) for each standard on low-concentration samples near the LOD (*n* = 10). This average area was converted to concentration calculated using the established linear response curve.

Limit of quantification (LOQ) were calculated by obtaining the average area (signal to noise ratio >10) for each standard on low-concentration samples near the LOD (*n* = 10). This average area was converted to concentration calculated using the established linear response curve. The LOD and LOQ are listed in Table 1.

##### Method Precision

To determine the intra-day (repeatability) precision of the rapid method, ‘Fuji’ apple lyophilised powder (*n* = 6) was analysed on three separate days. One-way analysis of variance (ANOVA) was used to calculate repeatability precision.

##### Method Trueness

Estimation of trueness was investigated by the recovery of (–)-epicatechin and procyanidin B2 in the fortified ‘Fuji’ apple lyophilised powder with average amounts of the flavan-3-ols and procyanidins. Apple powder were fortified to obtain samples containing approximately 20% of the expected content adding the known amounts of (–)-epicatechin and procyanidin B2 standards. Six fortified samples were prepared and analysed, as well as six unfortified samples. The mean concentrations of the fortified samples were referred to the amount added to the samples.

### 2.7. Quantification of the Flavan-3-ols and Procyanidins in Apples

Quantification was accomplished with the external standard approach. The concentrations of the flavan-3-ols and procyanidins in apple extract were equated by means of the calibration curves of (–)-epicatechin and procyanidin B2, respectively. Then, the contents of the flavan-3-ols and procyanidins in the apple were calculated as the whole apple flesh and peel accounted for 85% and 7% of the total apple weight, respectively, based on the Standard Tables of Food Composition in Japan of Ministry of Education, Culture, Sports, Science and Technology. Finally, the concentration of flavan-3-ols and procyanidins in the apple flesh with and without peel were expressed as mg per 100 g of fresh weight (FW).

### 2.8. Statistical Analysis

All data were presented as means ± standard deviation, where the statistical analysis of the apple weight, soluble solid, flavan-3-ols, and procyanidins over the years was performed using a one-way ANOVA followed by Tukey’s multiple comparisons test. In addition, the statistical analysis of flavan-3-ols and procyanidins in the ‘Fuji’ apple with the bagging treatment as well as in the apples with and without the peel in 2017 season were performed using an unpaired two-tailed Student’s t-test. All the graphs were produced using the GraphPad Prism^®^ software v8 for MAC OS X (GraphPad Software, San Diego, CA, USA, www.graphpad.com).

## 3. Results and Discussion

### 3.1. HPLC with Fluorescence Detection Was Used to Analyse the Flavan-3-ols and Procyanidins

#### 3.1.1. Optimized Method for the Analysis of Flavan-3-ols and Procyanidins in Apples

**Figure 1:**
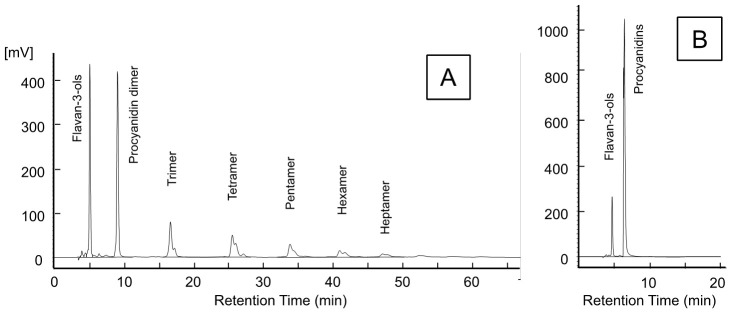
Normal-phase high-performance liquid chromatography profiles that were detected with a fluorescence emission of the ‘Fuji’ apple extract without peel by a (**A**) conventional and a (**B**) rapid quantitative methods. Analysis of flavan-3-ols and procyanidins by the conventional normal-phase chromatography (**A**) was performed by the method of Obara et al. [10].

Flavan-3-ols and procyanidins are abundantly found in food, including fruits, vegetables, nuts, seeds, and bark as well as beverages, including red wine and cocoa [30]. Normal-phase HPLC with fluorescence detection has been applied to analyse flavan-3-ols and procyanidins, providing good resolution and higher selective detection according to the polymerization degree of the flavan-3-ols and procyanidins. Chromatographic conditions were established by the previous quantitative studies to achieve the separation and resolution of complex mixture of oligomeric procyanidins from monomer to decamer [26,27,30]. We also analysed the flavan-3-ols and procyanidins in apples according to the polymerization degree, referring to the method of multi-laboratory assessment by Robbins et al. [26] (See Section 2.6.1) [10]. However, individual oligomeric procyanidin standards were not commercially available, particularly procyanidins with polymerization degree greater than tetramer. Furthermore, it was difficult to prepare in-house the individual standards for calibration of analytical instrumentation and quantitative method. Additionally, running these analysis for flavan-3-ols and procyanidins in apple can be lengthy, reaching 77 min (Figure 1A).

Hence, we modified from the previous chromatographic condition and developed a rapid quantitative method using HPLC with fluorescence detection to determine flavan-3-ols and procyanidins in a large number of apples, which generated more informative results in a shorter span of time. Particularly, gradient conditions were modified from the previous gradient condition, so that procyanidins with polymerization degree greater than dimer were eluted earlier. A typical chromatogram of a ‘Fuji’ apple extract is shown in Figure 1B, where procyanidin oligomers were eluted as a single and well-resolved peak, which enabled a clear separation of flavan-3-ols and procyanidins with a total run time of 20 min, including a more time-efficient column reconditioning shorter time relative to the conventional method. Additionally, individual oligomer standards from dimers to heptamers prepared from apples had the approximately same RT, resulting single peaks (Figure S2).

#### 3.1.2. Method Validations

##### Calibration Conditions

Calibration curve was constructed for (–)-epicatechin and procyanidin B2 standards, respectively. The fluorescence detection response to (–)-epicatechin and procyanidin B2 were plotted against a series of concentrations, and linear correlations were given with correlation coefficients (Table 1). The calibration employed for (–)-epicatechin and procyanidin B2 was a linear regression with seven concentration points. Calibration ranges were set during the concentration, which the apple sample were in the middle range. Additionally, all flavan-3-ols and procyanidin oligomers do not have the same response factors for fluorescence detection. In the present study, flavan-3-ols and procyanidins were equated to the standard of (–)-epicatechin and procyanidin B2, respectively.

##### Method Precision

Precision was expressed in terms of relative standard deviation (RSD) of the flavan-3-ols and procyanidins, which were below 3.97% and 2.91%, respectively. Thus, these data showed that the developed HPLC method enabled a highly accurate and precise measurement of flavan-3-ols and procyanidins in apple samples.

##### Method Trueness

Estimation of trueness was determined by fortifying an apple lyophilised powder with known amounts of (–)-epicatechin and procyanidin B2 standards. Trueness was expressed as% recovery of the analytes. The trueness of the flavan-3-ols and procyanidins were 101.0% and 97.6%. and the present results were consistent with previous reports of high recovery of monomeric flavan-3-ol and procyanidin dimer. However, the limitation of trueness in the method was the lack of the recovery tests of each individual oligomeric procyanidins from the apple samples. Hollands et al. reported that the recovery of the procyanidins with higher polymerization degree was observed to decrease [27].

### 3.2. Flavan-3-ol and Procyanidin Concentrations in the ‘Fuji’ Apples

Over 50% of the entire Japanese apple production takes place in the Aomori Prefecture. ‘Fuji’, ‘Tsugaru’, ‘Orin’, and ‘Jonagold’ apples are the varieties mainly produced, accounting for approximately 80% of the apple production in Japan [31]. ‘Fuji’ apple production is the largest in Japan, which accounts for approximately 53% of the total apple production in the 2018 harvest, because they are very sweet, juicy, medium- to large-sized, red striped, and highly stable. In Japan, ‘Fuji’ is cultivated both without bagging and with bagging to prevent diseases and achieve high storage stability. Therefore, we analysed flavan-3-ol and procyanidin concentrations in non-bagged and bagged ‘Fuji’ apples, separately.

Apple weights, soluble solids, flavan-3-ol and procyanidin concentrations in the non-bagged ‘Fuji’ in the 2016 to 2018 seasons have been summarised in Table 2. Fruit weight varied between 339.28 ± 88.67 g to 360.2 ± 87.42 g per fruit, while the level of soluble solids varied between 13.78 ± 0.94 to 14.18 ± 0.68 during the 2016 to 2018 seasons. Moreover, the content of flavan-3-ols ranged from 4.33 ± 1.06 mg/100 g FW in 2018 as well as to 6.72 ± 1.26 mg/100 g FW in 2017. In addition, the content of procyanidins ranged from 34.70 ± 6.15 mg/100 g FW in 2017 as well as to 37.77 ± 6.79 mg/100 g FW in 2018, where the ratio of procyanidins to flavan-3-ols (P/F ratio) was between 5.25 to 8.72.

Similarly, we evaluated the bagging of ‘Fuji’ apples during the 2016 to 2018 seasons (Table 3). Apple weights and soluble solids of bagged ‘Fuji’ apples before the CA storage were between 345.9 ± 87.40 g to 355.0 ± 86.34 g as well as from 12.87 ± 0.80 to 13.49 ± 0.99, respectively, while the concentrations of flavan-3-ols and procyanidins in the bagged ‘Fuji’ apples during the 2016 to 2018 seasons ranged from 4.70 ± 1.13 to 5.84 ± 1.11 mg/100 g FW and from 37.76 ± 6.99 to 39.18 ± 6.73 mg/100 g FW, respectively. Using normal phase HPLC, Gu et al. showed that flavan-3-ols and procyanidins of the ‘Fuji’ with peel were 6.46 and 63.13 mg/100 g FW, respectively [30], while our previous study investigated flavan-3-ols and procyanidins concentrations in 30 varieties of commercially-obtained and experimentally-harvested apples, where flavan-3-ols and procyanidins concentrations without the peel were 3.44 ± 0.42 and 29.34 ± 2.71 mg/100 g FW for ‘Orin’ apples as well as 5.70 ± 0.98 and 34.45 ± 6.96 mg/100 g FW for the non-bagged ‘Fuji’ apples, respectively, in the Aomori Prefecture during 2011 to 2013 [10].

Additionally, we evaluated the effects of CA storage on flavan-3-ols and procyanidins in the bagged ‘Fuji’ apples during the 2016 to 2017 seasons (Table 3). After the CA storage of four months, the contents of flavan-3-ols and procyanidins ranged from 5.03 ± 1.00 to 6.07 ± 1.12 mg/100 g FW as well as from 35.54 ± 7.49 to 41.63 ± 7.06 mg/100 g FW, respectively. The changes in the concentrations were slight, while the concentrations of flavan-3-ols and procyanidins were altered during the CA storage. Golding et al. [20] and Burda et al. [15] reported that the procyanidins in the peel of three other apple cultivars (Granny Smith, Lady Williams, and Crofton) remained relatively constant during the cold storage period. In contrast, the concentrations of flavan-3-ols were increased during the first few month storage period and then remained constant or slightly declined. Similar results were also obtained on the flesh (cold storage conditions at 2 °C of usually for marketing for 4 month) by Napolitano et al. [24]. The increase of polyphenols was related to the ripening due to the ethylene hormone action during the post-harvest treatment. Ethylene production in ‘Fuji’ apples might be lower at the CA storage condition [32].

**Figure 2:**
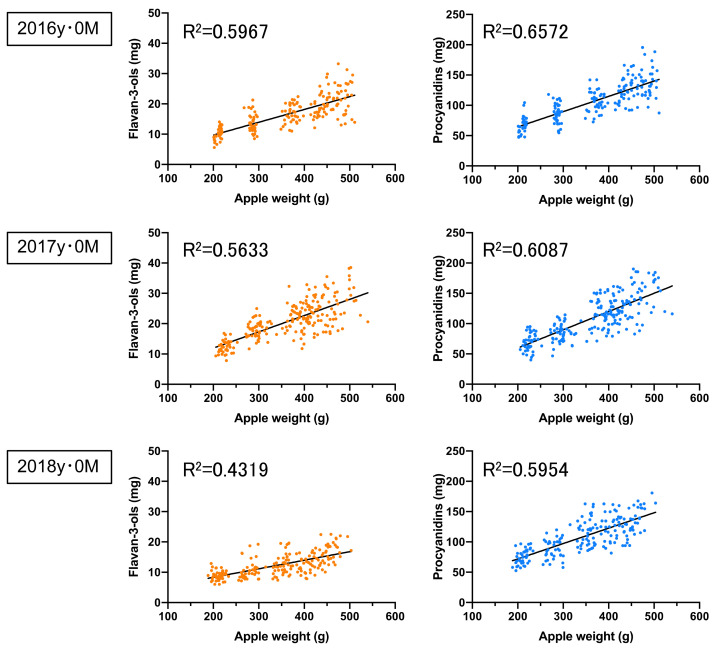
The correlations between the apple weights and both the flavan-3-ol and the procyanidin concentrations in the non-bagged ‘Fuji’ apples. The apples were analysed in 2016y·0M (*n* = 196), 2017y·0M (*n* = 219), and 2018y·0M (*n* = 194), where y refers to the year, M refers to the month, and n refers to the number of samples.

**Figure 3:**
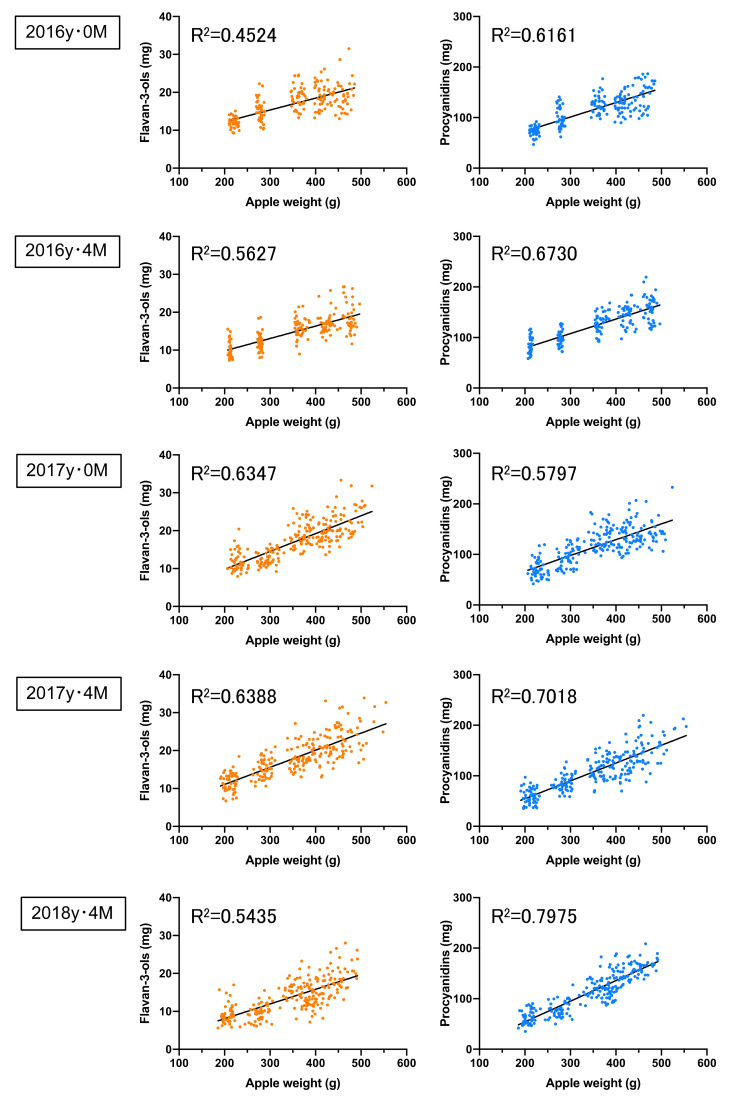
The correlations between the apple weights and both the flavan-3-ol and the procyanidin concentrations in the bagged ‘Fuji’ apples. The apples were analysed in 2016y·0M (*n* = 195), 2016y·4M (*n* = 199), 2017y·0M (*n* = 235), 2017y·4M (*n* = 235), and 2018y·4M (*n* = 237), where y refers to the year, M refers to the month, and *n* refers to the number of samples.

Furthermore, the correlations between the apple weight and both the flavan-3-ols and the procyanidins per apple in the non-bagged ‘Fuji’ during the 2016 to 2018 season were relatively high, with a correlation coefficient of 0.4319 to 0.5967 and 0.5954 to 0.6572, respectively (Figure 2). Similarly, the correlations between apple weight and both the flavan-3-ols and the procyanidins per apple in the bagged ‘Fuji’ during the 2016 to 2018 season were relatively high, with a correlation coefficient of 0.4524 to 0.6388 and 0.5797 to 0.7975, respectively (Figure 3). In contrast, no correlation (*R^2^* = 0.0043 to 0.1497, 0.0002 to 0.0664, respectively) was found between the apple soluble solids relative to flavan-3-ols and procyanidins in non-bagged and bagged ‘Fuji ’apples (Figures S3 and S4). In general, flavan-3-ols and procyanidins have been reported to decrease with apple fruit development and ripening [33].

Moreover, flavan-3-ol concentration in the bagged and non-bagged ‘Fuji’ apples was significantly different due to the bagging treatment (Figure S5). Similarly, procyanidins contents in the bagged ‘Fuji’ apples were significantly higher relative to the non-bagged ‘Fuji’ apples during the 2016 to 2017 seasons (*p* < 0.001). Generally, apple fruit bagging treatment decrease most polyphenol concentrations in the peel and flesh. In the peel, anthocyanins were the most sensitive to the bagging treatment. Chen et al. also reported that the bagging treatment until harvest clearly lowered the concentration of flavan-3-ols and procyanidins in the peels of ‘Golden Delicious’, ‘Red Delicious’, and ‘Royal Gala’ apples [34]. In Japan, the bagging treatment of the ‘Fuji’ apples consisted of bagging up to 30 days before harvest, which enhanced the biosynthesis of anthocyanin in the peel of the ‘Fuji’ apples [35]. Therefore, the bagging treatment might have an effect on flavan-3-ols and procyanidins biosynthesis or the promotion by sunlight irradiation and temperature. Hence, cultivation studies of ‘Fuji’ apples under controlled sunlight exposure due to the bagging treatment are required to determine whether the bagging treatment has an effect on the biosynthesis of flavan-3-ols and procyanidins by the cultivation methods. These effects can include tree shape and rootstock that may be affected by ultraviolet irradiation and would alter the amount of procyanidins in apples. One of the limitations of the present study is that it was unclear how the bagging treatment affects the amount of flavan-3-ols and procyanidins via the amount of sunlight received and hormones, because apple samples were not cultivated in the experimental field. Further investigation is required into the regulation of flavan-3-ols and procyanidins in apple.

### 3.3. Flavan-3-ol and Procyanidin Concentrations in the ‘Orin’ Apples

**Figure 4:**
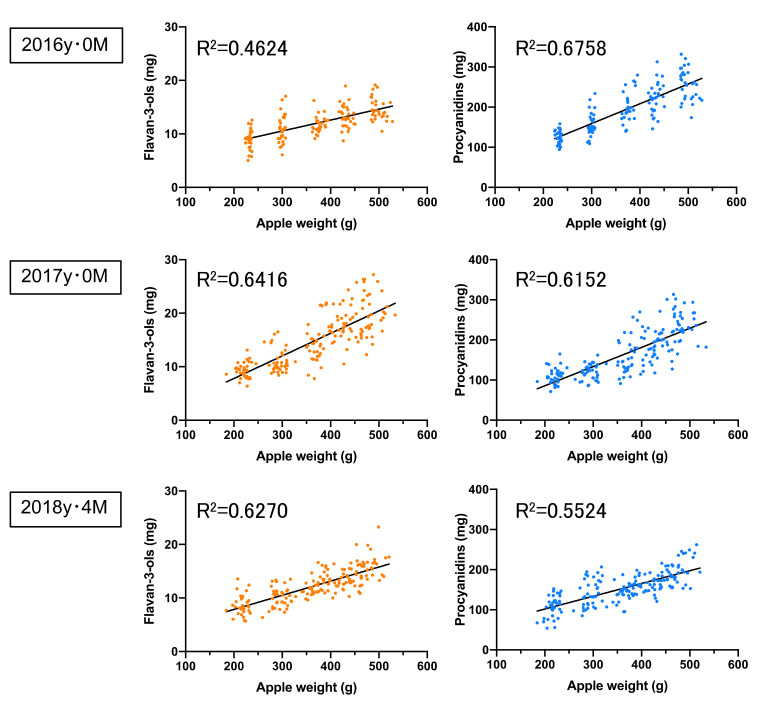
The correlations between the apple weights and both the flavan-3-ol and the procyanidin concentrations in the non-bagged ‘Orin’ apples. The apples were analysed in 2016y·0M (*n* = 148), 2017y·0M (*n* = 177), and 2018y·4M (*n* = 179), where y refers to the year, M refers to the month, and n refers to the number of samples.

Next, we evaluated the ‘Orin’ apples during the 2016 to 2018 seasons (Table 4). ‘Orin’ apples with a yellow or green skin colour were bred between ‘Indo’ and ‘Golden Delicious’, which are one of the most popular apples in Japan. Fruit weight varies between 356.8 ± 93.25 g to 368.7 ± 94.53 g per fruit, while the level of soluble solids varies between 14.12 ± 0.61 to 14.20 ± 0.83 during the 2016 to 2018 seasons. In addition, the weight and soluble solids of the ‘Orin’ apples were slightly higher than those of the ‘Fuji’ apples, where the concentrations of flavan-3-ols and procyanidins ranged from 3.92 ± 0.84 to 4.75 ± 0.93 as well as and from 51.10 ± 10.79 to 61.79 ± 9.95 mg/100 g FW, respectively. Furthermore, the P/F ratio was observed to be 11.17 to 15.76 in the ‘Orin’ apples, since they contained less flavan-3-ols and more procyanidins relative to the ‘Fuji’ apples. Additionally, the correlations between the apple weight and both the flavan-3-ols and the procyanidins per apple in the ‘Orin’ during the 2016–2018 seasons were relatively high, with a correlation coefficient of 0.4624 to 0.6416 and 0.5524 to 0.6758, respectively (Figure 4). Similarly, no correlation (R^2^ = 0.0012 to 0.0668, 0.0236 to 0.1368, respectively) was found between the apple soluble solids relative to flavan-3-ols and procyanidins per ‘Orin’ apples (Figure S6).

### 3.4. Differences in the Flavan-3-ols and Procyanidins in the Apple Peel

**Figure 5:**
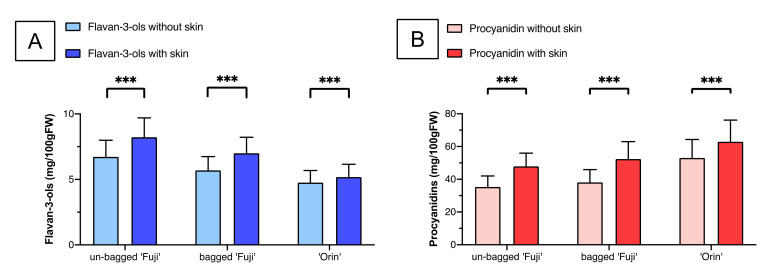
Comparison of the (**A**) flavan-3-ols and (**B**) procyanidins in the ‘Fuji’ and ‘Orin’ apples with and without the peel during the 2017 season. Data are shown as means ± deviation, where significant differences were evaluated using an unpaired two-tailed Student’s *t*-test (*** *p* < 0.001).

Compared to the apple flesh, the apple peel contains more polyphenols with different profiles [17,36]. The peel also contains cyanidin glycosides, quercetin glycosides, phloretin glycosides as well as hydroxycinnamic acids, flavan-3-ols, and procyanidins. However, how much the apple flesh contains relative to the peel in terms of flavan-3-ols and procyanidins was unknown. During the 2017 season, we evaluated the contents of flavan-3-ols and procyanidins with or without the peel in the un-stored apples, in which the non-bagged ‘Fuji’ with the peel contained flavan-3-ol and procyanidin contents of 8.22 ± 1.47 and 47.93 ± 8.06 mg/100 g FW, respectively. Similarly, the bagged ‘Fuji’ with peel contained 6.99 ± 1.22 and 52.37 ± 10.70 mg/100 g FW, respectively (Figure 5). As found in the previous reports, we observed an increase in the contents of flavan-3-ols and procyanidins in the non-bagged and bagged ‘Fuji’ apples with the peel, which were 22.3% and 35.9% higher than those without the peel. Similarly, ‘Orin’ apples contained 9.1% and 18.6% more flavan-3-ols and procyanidins, respectively, but did not show as much difference as the ‘Fuji’ with and without the peel. There are many reports recommending eating the skin because it contains a lot of polyphenols. However, the skin is only about 7% of the total fruit, and consumers may be misrepresented that they only need to eat the skin. In Japan, people prefer to eat apples without the peel, with the exception of processed products, such as juices. The present data showed that the consumption of the apple flesh with the peel allows the efficient ingestion of flavan-3-ols and procyanidins to obtain their health benefits.

Various physiological functions of flavan-3-ols and procyanidins have been reported [37], where the data strongly suggest that they play an important part in the mechanisms underlying various health benefits. Moreover, epidemiological studies have an important role in accurately assessing dietary intake, which provide long-term data from a large population to evaluate the relationship between disease and diet. The assessment of dietary intake would depend on self-reported data, such as food-frequency questionnaires, and food composition databases, such as the United States Department of Agriculture [38] and the Phenol Explorer [39]. Various studies have also estimated the intake of flavonoids, including flavan-3-ols and procyanidins in the United States and in the European Union [40,41]. In the European Union, the total intake of flavan-3-ols and procyanidins were estimated to be 77.0 and 123.4 mg/day, respectively, where one of the major food sources were fruits, which included apples. In Japan, there are no databases of flavonoids and polyphenols that include dietary flavan-3-ols and procyanidins. Therefore, the results of this study, although the data were limited in terms of apple varieties and production areas, would be able to estimate the intake of flavan-3-ols and procyanidins from apples. The amounts of apple products, including the apple processed products, consisted of 756,000 tons in the 2018 season [42], with an apple intake per Japanese individual of 20.8 g/day [43]. Although the estimated amount of flavan-3-ols and procyanidins varies depending on whether or not the peel is included, the daily intake from apples of flavan-3-ols and procyanidins by the Japanese people were estimated to be 0.82~1.40 and 7.22~12.36 mg, respectively. Since procyanidins are predominant in modern diets, including those found in fruits, cacao, tea, and red wine, to name a few, it is important to further clarify the flavan-3-ol and procyanidin contents in the Japanese diet and to estimate the intake of flavan-3-ols and procyanidins from Japanese foods. Additionally, a new food labelling system for food with function claims was started in Japan [44]. Under this system, industry and agricultural producers independently should evaluate scientific evidence on agricultural foods, describe the functional properties, and indicate the content of the functional ingredients for the consumers. So, the present results also would be able to estimate the differences of flavan-3-ols and procyanidins from apples.

## 4. Conclusions

In the present study, we established a rapid quantitative HPLC method with fluorescence detection to examine the flavan-3-ol and procyanidin contents, which were observed in the non-bagged and bagged ‘Fuji’ and ‘Orin’ apples and are summarised in Table 2, Table 3 and Table 4. We found that the procyanidin contents in the ‘Orin’ apples were higher relative to the ‘Fuji’ apples, while the flavan-3-ols contents were similar. In the ‘Fuji’ apples, the procyanidins contents were significantly increased during every season from 2016 to 2018 by the bagging treatment, while only slight changes were observed in the flavan-3-ols and procyanidins during the CA storage.

## Figures and Tables

**Table 1:** Parameters of the analytical method of the flavan-3-ols and procyanidins.

Standard Compounds	Retention Time (min)	Calibration Range (µg/mL)	Calibration Formula	Regression Factor (R^2^)	RSD (%)	LOD/LOQ
(–)-Epicatechin	4.56	4.5–45.0	Y = 269140X + 34947	0.9989	3.97	0.007/0.021
Procyanidin B2	6.12	10.55–105.5	Y = 205281X + 419517	0.9987	2.91	0.010/0.030

Calibration curves of the flavan-3-ols and procyanidins were prepared and obtained from (-)-epicatechin and procyanidin B2 standards, respectively.

**Table 2:** Concentrations of the flavan-3-ols and procyanidins in the non-bagged ‘Fuji’ apples that were harvested during the 2016–2018 seasons.

Seasons	Storage Periods (months)	*n*	Weight (g)	Soluble Solid (°Brix)	Flavan-3-ols (mg/100 g FW)	Procyanidins (mg/100 g FW)	P/F Ratio
2016	0	196	357.9 ± 99.23 ^a^	13.78 ± 0.94 ^a^	5.42 ± 1.09 ^a^	34.70 ± 6.15 ^a^	6.40
2017	0	219	360.2 ± 87.42 ^a^	14.18 ± 0.68 ^b^	6.72 ± 1.26 ^b^	35.27 ± 6.73 ^a^	5.25
2018	0	194	339.8 ± 88.67 ^a^	14.11 ± 1.01 ^b^	4.33 ± 1.06 ^c^	37.77 ± 6.79 ^b^	8.72

Values are means ± standard deviation. P/F refers to the procyanidins to flavan-3-ols ratio. Flavan-3-ols and procyanidins were equated to (–)-epicatechin and procyanidin B2, respectively. The mean values within a verse with different letters are significantly different at *p* < 0.05 according to Tukey’s multiple comparisons test.

**Table 3:** Concentrations of the flavan-3-ols and procyanidins in the bagged ‘Fuji’ apples that were harvested during the 2016–2018 seasons.

Seasons	Storage Periods (months)	*n*	Weight (g)	Soluble Solid (°Brix)	Flavan-3-ols (mg/100 g FW)	Procyanidins (mg/100 g FW)	P/F Ratio
2016	0	195	345.9 ± 87.40 ^a^	13.49 ± 0.99 ^a^	5.84 ± 1.11 ^a^	39.18 ± 6.73 ^a^	6.71
	4	199	351.7 ± 96.24 ^a^	12.97 ± 0.52 ^b^	5.03 ± 1.01 ^b^	41.63 ± 7.06 ^b^	8.28
2017	0	235	355.0 ± 86.34 ^a^	13.39 ± 0.95 ^a^	5.69 ± 1.06 ^a^	38.13 ± 7.75 ^a^	6.71
	4	235	349.3 ± 94.76 ^a^	12.87 ± 0.80 ^b^	6.06 ± 1.12 ^c^	35.74 ± 7.45 ^c^	5.86
2018	4	237	336.7 ± 88.06 ^a^	13.38 ± 0.88 ^c^	4.70 ± 1.13 ^b^	37.76 ± 6.99 ^a^	8.03

Values are means ± standard deviation. P/F refers to the procyanidins to flavan-3-ols ratio. Flavan-3-ols and procyanidins were equated to (–)-epicatechin and procyanidin B2, respectively. The mean values within a verse with different letters are significantly different at *p* < 0.05 according to Tukey’s multiple comparisons test.

**Table 4:** Concentrations of the flavan-3-ols and procyanidins in non-bagged ‘Orin’ apples that were harvested during the 2016–2018 seasons.

Seasons	Storage Periods (months)	*n*	Weight (g)	Soluble Solid (°Brix)	Flavan-3-ols (mg/100 g FW)	Procyanidins (mg/100 g FW)	P/F Ratio
2016	0	148	368.7 ± 94.53 ^a^	14.12 ± 0.61 ^a^	3.92 ± 0.84 ^a^	61.79 ± 9.95 ^a^	15.76
2017	0	177	361.4 ± 94.42 ^a^	14.19 ± 0.80 ^a^	4.75 ± 0.93 ^b^	53.05 ± 11.25 ^b^	11.17
2018	4	179	356.8 ± 93.25 ^a^	14.20 ± 0.83 ^a^	4.04 ± 0.76 ^a^	51.10 ± 10.79 ^b^	12.65

Values are means ± standard deviation. P/F refers to the procyanidins to flavan-3-ols ratio. Flavan-3-ols and procyanidins were equated to (–)-epicatechin and procyanidin B2, respectively. The mean values within a verse with different letters are significantly different at *p* < 0.05 according to Tukey’s multiple comparisons test.

## Data Availability

Most data presented in this study are in the Supplementary Materials. The remaining data are available on request from the corresponding author.

## Supplementary Materials

The following are available online at https://www.mdpi.com/2304-8158/10/2/274/s1, Figure S1: Chemical structures of flavan-3ols and procyanidins in apples, Figure S2: Chromatograms of each individual standards and apple extract by the rapid method. Figure S3: The correlations between apple soluble solid and flavan-3-ols and procyanidins concentrations in unbagged ‘Fuji’ apples. Figure S4: The correlations between apple soluble solid and flavan-3-ols and procyanidins concentrations in bagged ‘Fuji’ apples. Figure S5: The correlations between apple soluble solid and flavan-3-ols and procyanidins concentrations in unbagged ‘Orin’ apples. Figure S6: Comparison of the (A) flavan-3-ols and (B) procyanidins in the ‘Fuji’ apples with and without bagging during the 2016 and 2017 seasons.
