# Supplementary material for: The Differences in the Flavan-3-ol and Procyanidin Contents of the Japanese ‘Fuji’ and ‘Orin’ Apples Using a Rapid Quantitative High-Performance Liquid Chromatography Method: Estimation of the Japanese Intake of Flavan-3-ols and Procyanidins from Apple as Case Study
Source: Foods. 2021 Jan 29;10(2):274. doi: 10.3390/foods10020274 (PMC7911932; doi:10.3390/foods10020274)
Supplement: Supplementary file 1 [file foods-10-00274-s001.pdf]

Supplementary File

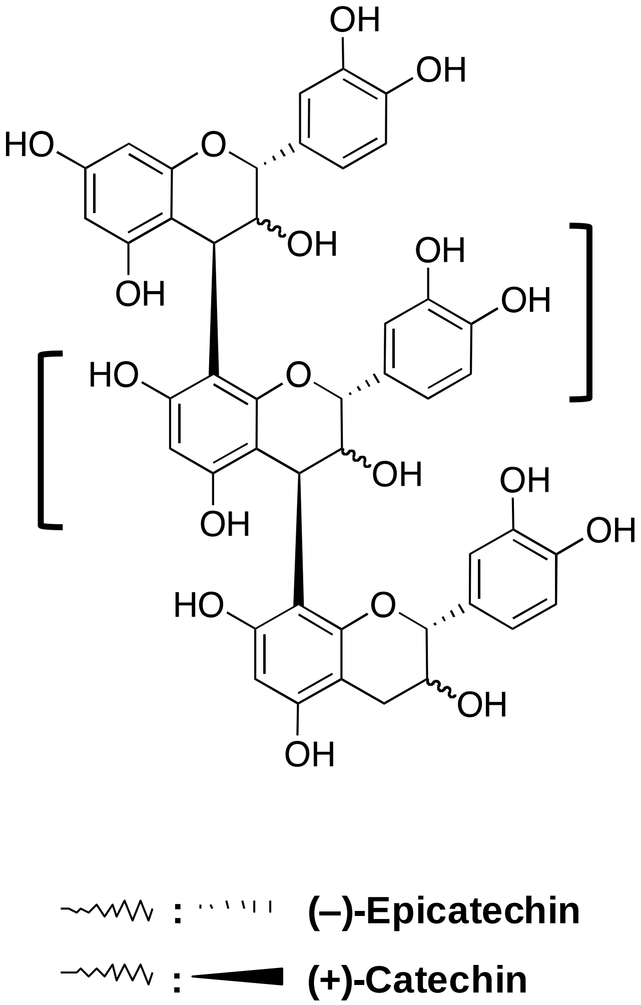

Figure S1. Chemical structures of flavan-3ols and procyanidins in apples.

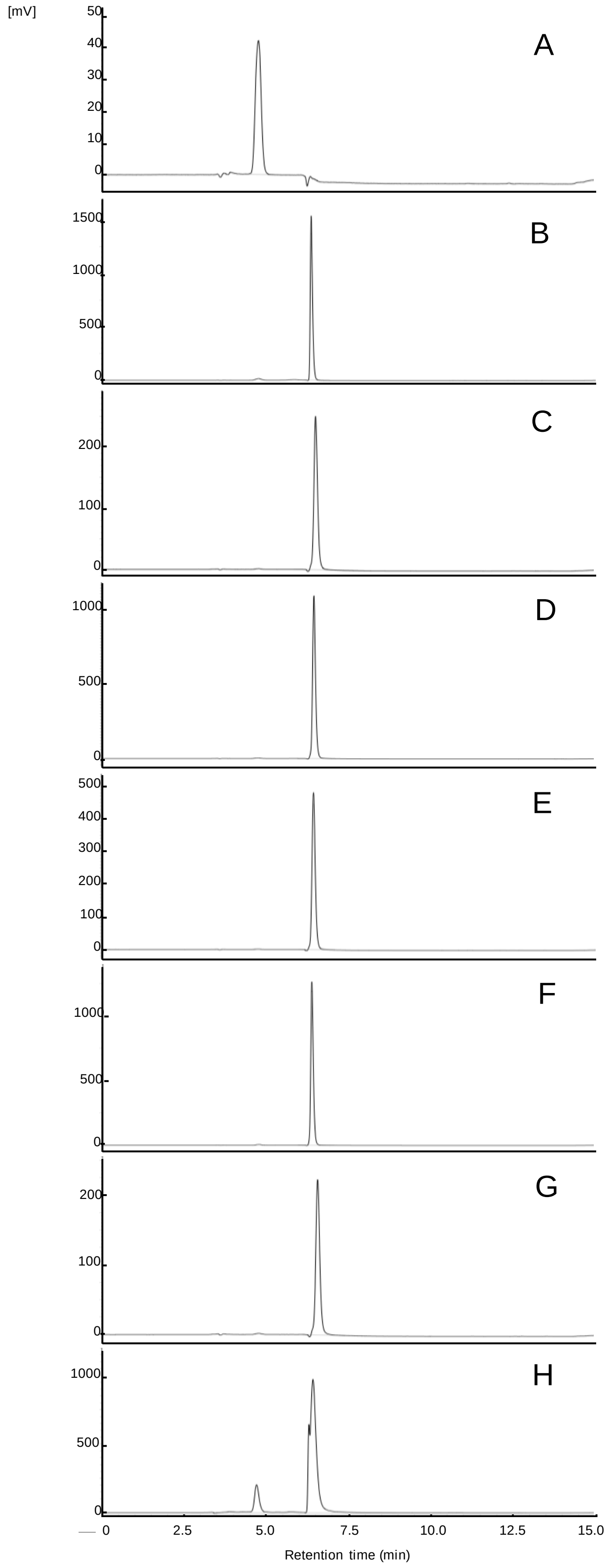

Figure S2. Chromatograms of each individual standards and apple extract by the rapid method. A, monomer; B, dimer; C, trimer; D, tetramer; E, pentamer; F, hexamer; G, heptamer, and H, apple extract from 'Fuji'. Each individual standard fractions were prepared by the preparative method.

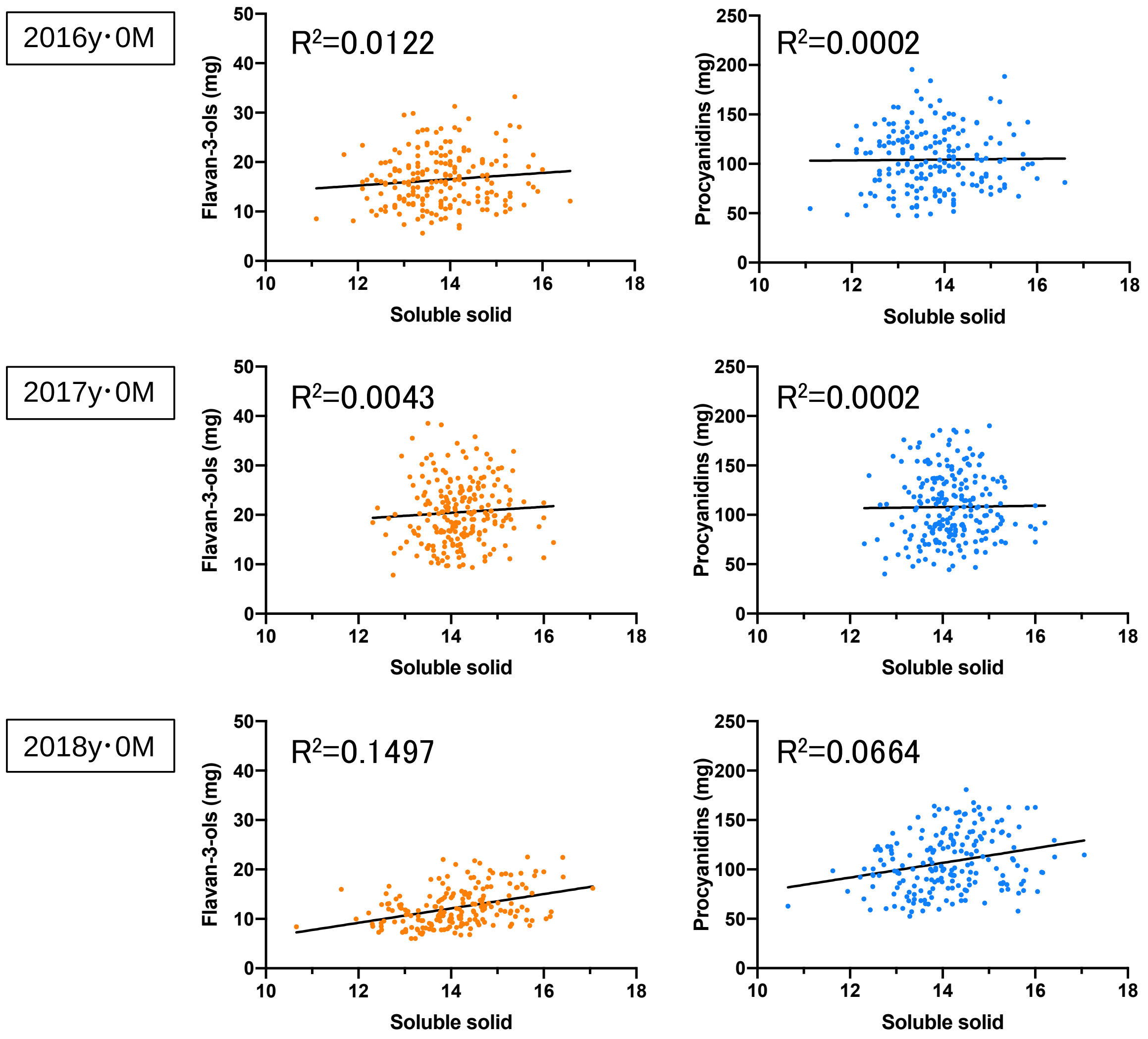

Figure S3. The correlations between apple soluble solid and flavan-3-ols and procyanidins concentrations in unbagged 'Fuji' apples. Apples were analyzed in 2016y･0M (n=196), 2017y･0M (n=219) and 2018y･0M (n=194).

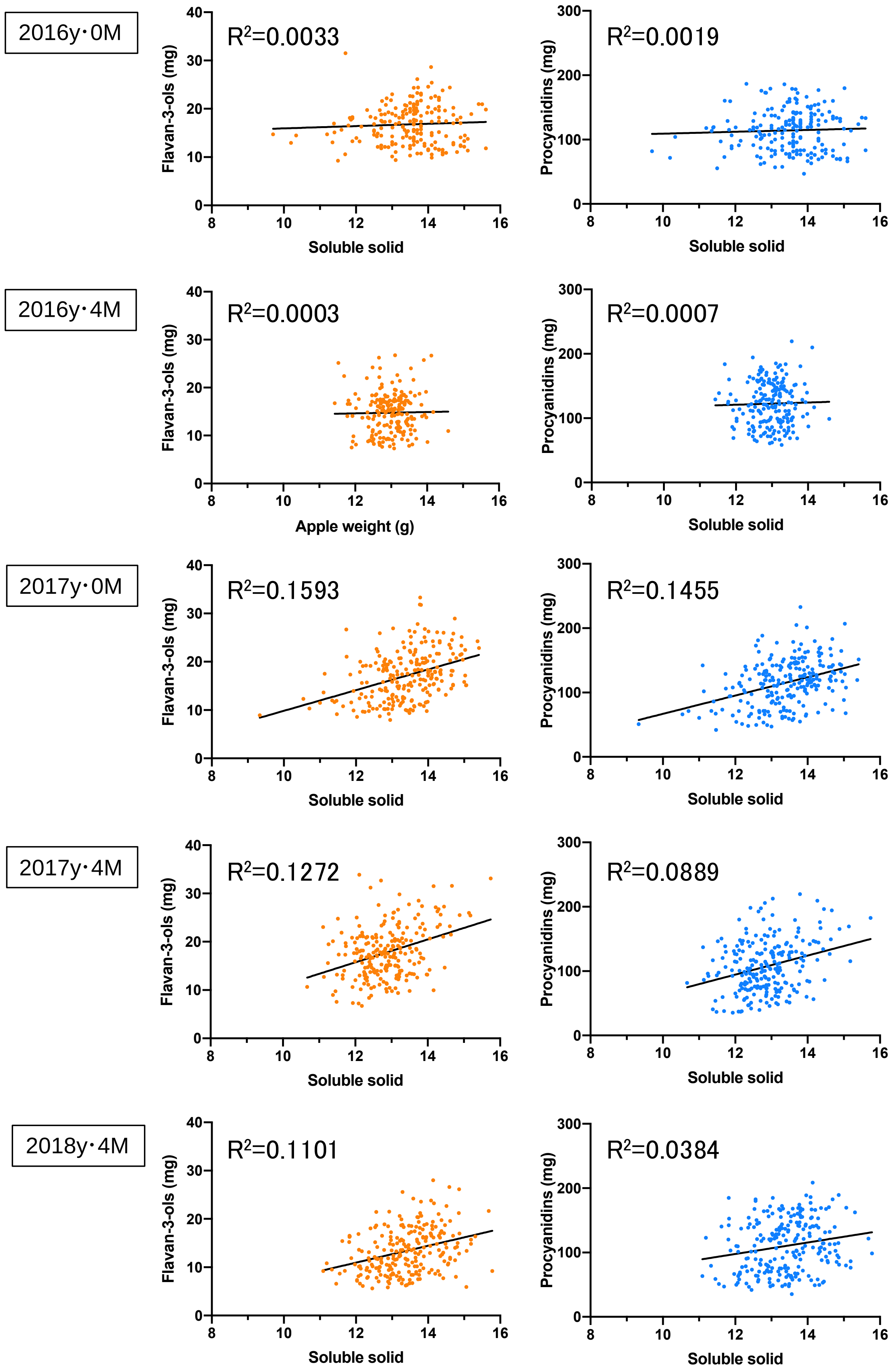

Figure S4 The correlations between apple soluble solid and flavan-3-ols and procyanidins concentrations in bagged 'Fuji' apples. Apples were analyzed in 2016y・0M (n=195), 2016y・4M (n=199), 2017y・0M (n=235), 2017y・4M (n=235) and 2018y・4M (n=237).

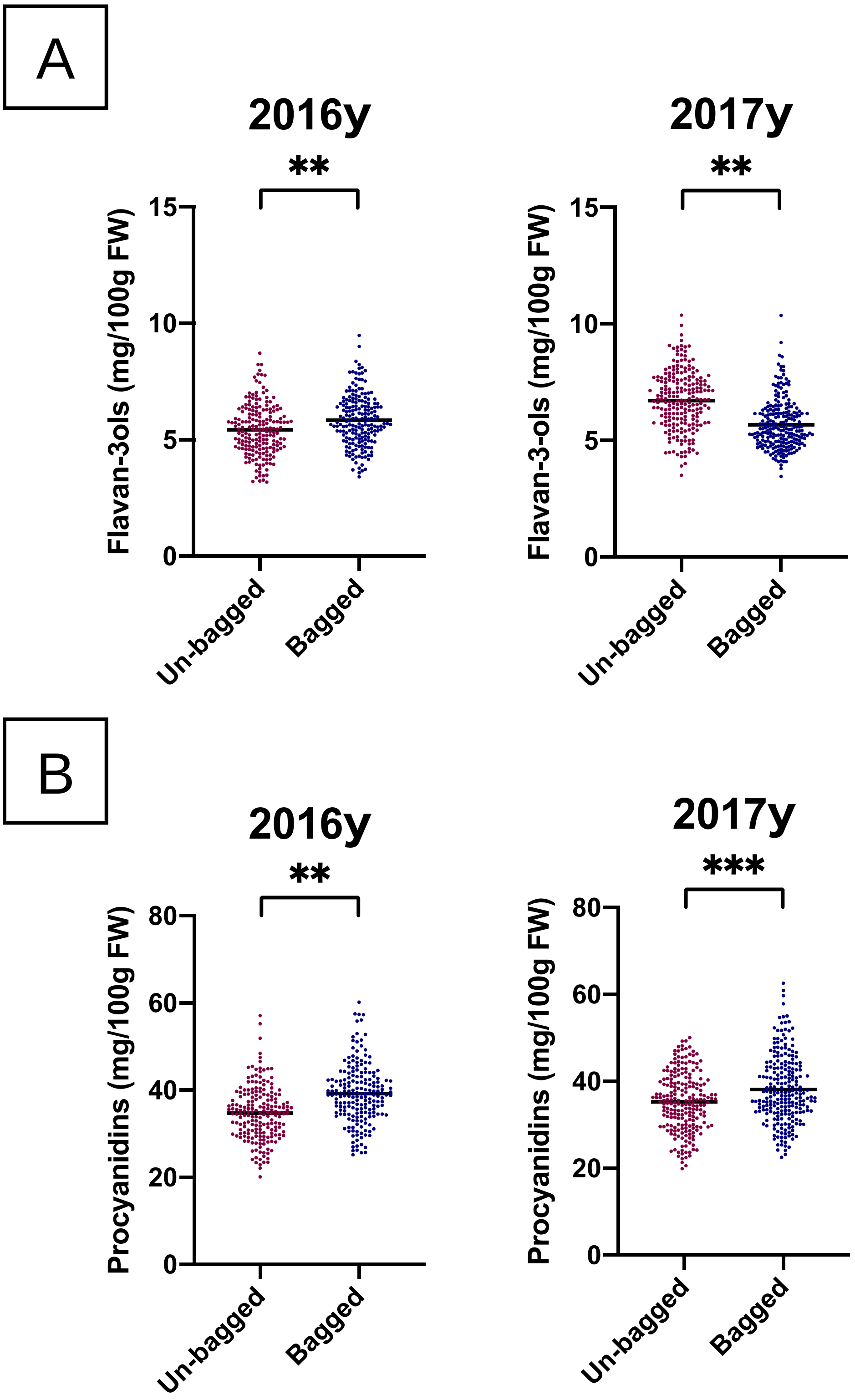

Figure S5. Comparison of the (A) flavan-3-ols and (B) procyanidins in the 'Fuji' apples with and without bagging during the 2016 and 2017 seasons. Data are shown as means ± deviation, where the significant differences were evaluated using an unpaired two-tailed Student's t-test (**: $p < 0.01$, ***: $p < 0.001$).

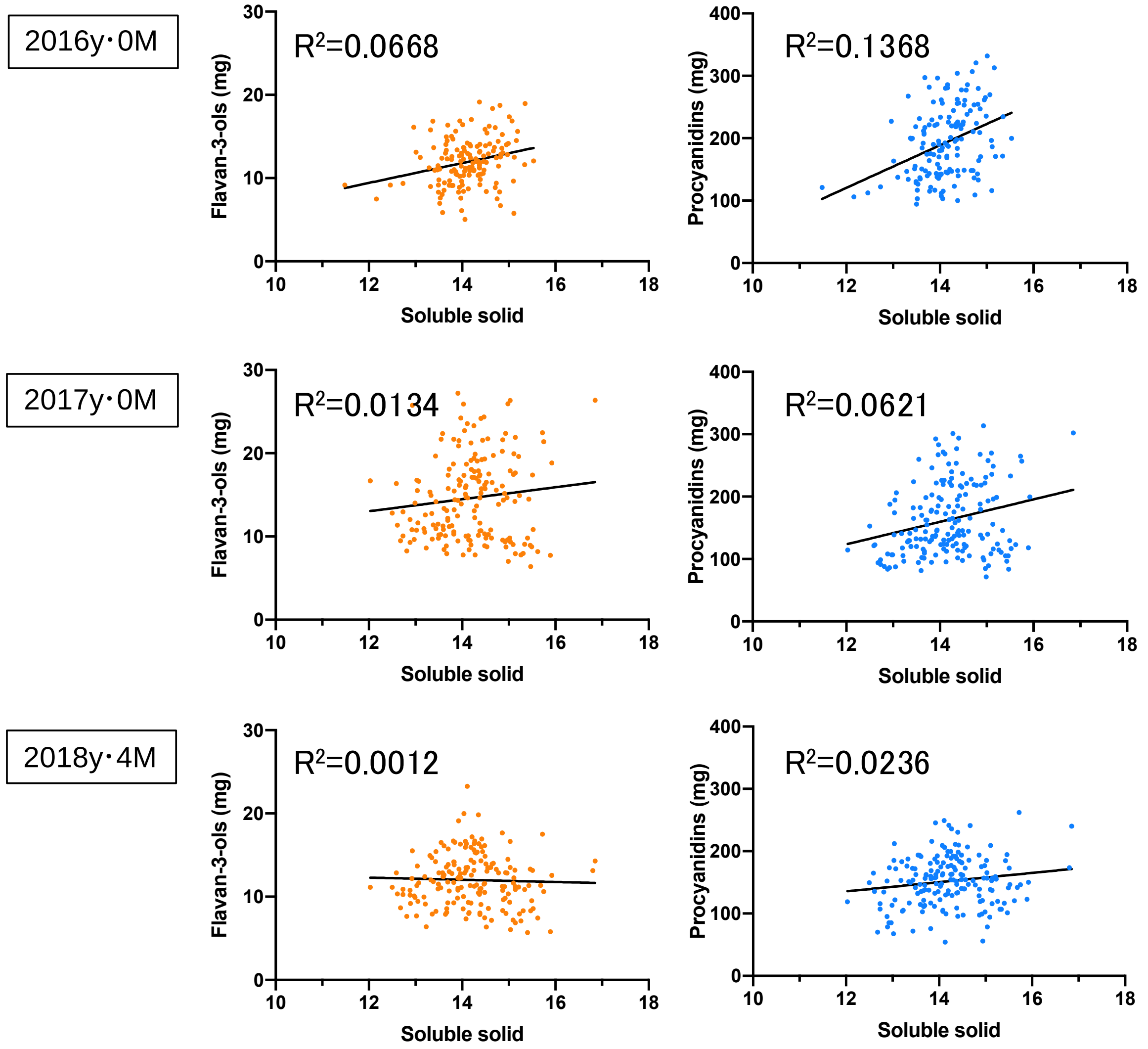

Figure S6. The correlations between apple soluble solid and flavan-3-ols and procyanidins concentrations in unbagged 'Orin' apples. Apples were analyzed in 2016y・0M (n=148), 2017y・0M (n=177) and 2018y・4M (n=179).
